# Racial/ethnic differences in multimorbidity development and chronic disease accumulation for middle-aged adults

**DOI:** 10.1371/journal.pone.0218462

**Published:** 2019-06-17

**Authors:** Ana R. Quiñones, Anda Botoseneanu, Sheila Markwardt, Corey L. Nagel, Jason T. Newsom, David A. Dorr, Heather G. Allore

**Affiliations:** 1 Department of Family Medicine, Oregon Health & Science University, Portland, Oregon, United States of America; 2 School of Public Health, Oregon Health & Science University, Portland, Oregon, United States of America; 3 Department of Health & Human Services, University of Michigan, Dearborn, Michigan, United States of America; 4 Institute of Gerontology, University of Michigan, Ann Arbor, Michigan, United States of America; 5 College of Nursing, University of Arkansas for Medical Sciences, Little Rock, Arkansas, United States of America; 6 Department of Psychology, Portland State University, Portland, Oregon, United States of America; 7 Department of Medical Informatics and Clinical Epidemiology, Oregon Health & Science University, Portland, Oregon, United States of America; 8 Department of Internal Medicine, School of Medicine, Yale University, New Haven, Connecticut, United States of America; 9 Department of Biostatistics School of Public Health, Yale University, New Haven, Connecticut, United States of America; Nathan S Kline Institute, UNITED STATES

## Abstract

**Background:**

Multimorbidity–having two or more coexisting chronic conditions–is highly prevalent, costly, and disabling to older adults. Questions remain regarding chronic diseases accumulation over time and whether this differs by racial and ethnic background. Answering this knowledge gap, this study identifies differences in rates of chronic disease accumulation and multimorbidity development among non-Hispanic white, non-Hispanic black, and Hispanic study participants starting in middle-age and followed up to 16 years.

**Methods and findings:**

We analyzed data from the Health and Retirement Study (HRS), a biennial, ongoing, publicly-available, longitudinal nationally-representative study of middle-aged and older adults in the United States. We assessed the change in chronic disease burden among 8,872 non-Hispanic black, non-Hispanic white, and Hispanic participants who were 51–55 years of age at their first interview any time during the study period (1998–2014) and all subsequent follow-up observations until 2014. Multimorbidity was defined as having two or more of seven somatic chronic diseases: arthritis, cancer, heart disease (myocardial infarction, coronary heart disease, angina, congestive heart failure, or other heart problems), diabetes, hypertension, lung disease, and stroke. We used negative binomial generalized estimating equation models to assess the trajectories of multimorbidity burden over time for non-Hispanic black, non-Hispanic white, and Hispanic participants. In covariate-adjusted models non-Hispanic black respondents had initial chronic disease counts that were 28% higher than non-Hispanic white respondents (IRR 1.279, 95% CI 1.201, 1.361), while Hispanic respondents had initial chronic disease counts that were 15% lower than non-Hispanic white respondents (IRR 0.852, 95% CI 0.775, 0.938). Non-Hispanic black respondents had rates of chronic disease accumulation that were 1.1% slower than non-Hispanic whites (IRR 0.989, 95% CI 0.981, 0.998) and Hispanic respondents had rates of chronic disease accumulation that were 1.5% faster than non-Hispanic white respondents (IRR 1.015, 95% CI 1.002, 1.028). Using marginal effects commands, this translates to predicted values of chronic disease for white respondents who begin the study period with 0.98 chronic diseases and end with 2.8 chronic diseases; black respondents who begin the study period with 1.3 chronic diseases and end with 3.3 chronic diseases; and Hispanic respondents who begin the study period with 0.84 chronic diseases and end with 2.7 chronic diseases.

**Conclusions:**

Middle-aged non-Hispanic black adults start at a higher level of chronic disease burden and develop multimorbidity at an earlier age, on average, than their non-Hispanic white counterparts. Hispanics, on the other hand, accumulate chronic disease at a faster rate relative to non-Hispanic white adults. Our findings have important implications for improving primary and secondary chronic disease prevention efforts among non-Hispanic black and Hispanic Americans to stave off greater multimorbidity-related health impacts.

## Introduction

Multimorbidity, defined as two or more co-occurring chronic diseases, is common in old age, highly disabling, and costly [1–3]. A growing body of work corroborates the compounding effects of multimorbidity on health outcomes beyond the risk attributable to individual diseases [4,5] and identifies specific racial and ethnic groups who are at greater risk of poor outcomes [6–8]. However, few studies have documented the onset and progression of multimorbidity for various population subgroups over time. Indeed, the U.S. Department of Health and Human Services reported on remaining gaps in clinical and research practice and highlighting the need to identify and understand disparities related to race and ethnicity [9,10].

Whereas the last 50 years has resulted in important longevity gains for older adults in the U.S. these improvements have not been universal. Slowed and even worsening trends for the most vulnerable older adults are evident, a regrettable and distinct trend that stands in contrast to other high-income countries [11]. Indeed, older at-risk adults—among them women, underrepresented racial and ethnic minorities, and adults with low educational attainment or low socioeconomic status—are living for extended periods of their lives in sicker and more disabled states and are at greatest risk for premature morbidity and mortality [12,13]. Despite some evidence that high chronic disease burden disproportionately impacts racial and ethnic minorities, it is unclear how chronic diseases accumulate differentially in mid- and late-life for distinct population groups.

Understanding how chronic diseases accumulate over time and develop into multimorbidity among and between adults from various racial and ethnic backgrounds is necessary to better plan and deliver care. Groups with earlier onset and/or faster chronic disease accumulation and multimorbidity development represent prime targets for programs and interventions aimed at limiting the impact of co-occurring diseases on other outcomes. This is particularly relevant for racial and ethnic minority adults, who have lower rates of insurance coverage prior to reaching Medicare eligibility age [14,15] and also have lower rates of utilization of preventive and diagnostic services even after becoming eligible for Medicare coverage [16,17], suggesting a higher burden of unmet morbidity needs in these populations.

This study represents a logical continuation of our earlier work [6]. In order to better understand the process of developing multimorbidity and the accumulation of chronic disease burden over time, we undertook the present analysis, which focuses on the earliest age in which we observe study participants and follow these participants over all available time points. To this end, the aim of this study is to evaluate how multimorbidity develops and progresses over time among middle-aged non-Hispanic black, non-Hispanic white, and Hispanic Americans. Ensuring that treatment guidelines and disease management programs address the impact of multimorbidity on the most complex and vulnerable patients represents a challenge and a charge to healthcare systems worldwide.

## Methods

### Data

The Health and Retirement Study (HRS) is a nationally-representative, longitudinal cohort study of non-institutionalized middle- and older-aged adults which explores the transitions in health that occur toward the end of a person’s work life and into retirement and permit a wide array of investigations involving sociodemographic factors as well as patient-reported outcomes that are generalizable to the U.S. population of middle-aged and older adults [18]. The HRS is supported by the National Institute on Aging and the Social Security Administration and as such, represents an important source of publicly-available data for health-related research. Respondents and their partners are assessed every two years from the time of their entry into the survey until death. The initial HRS cohort was surveyed in 1992, with additional cohorts added in 1993, 1998 and every six years onward to refresh the sample and ensure ongoing sample representativeness [19]. The present study utilized all available biennial assessments for birth cohorts between 1998 through 2014, the most recent finalized data collection available to date [20].

### Study population

This study followed participants over time at the earliest study-entry eligible age for the longest time possible and with consistent chronic disease burden reporting. This approach maximized our ability to capture chronic disease accumulation and multimorbidity onset over an extended period. The study sample was restricted to respondents whose first interview occurred between the ages of 51 and 55 years old. 31,501 HRS respondents were interviewed between 1998 and 2014, were living in the community, and were age eligible for the study (i.e., respondents with a positive survey weight). Of these respondents, 10,126 were first interviewed at age 51–55 years old. Consistent with prior publications [21], 844 respondents with clinically-inconsistent self-reporting of chronic disease were excluded (demographic characteristics for these excluded respondents are reported in the **Table B in S1 Appendix**); 33 respondents with missing responses across all chronic conditions at their first age-eligible interview were excluded; and 8 respondents without race/ethnicity information were excluded. Additionally, we excluded 369 respondents that identified as American Indian, Asian or “Other” due to small and heterogeneous racial and ethnic categories that would have insufficient statistical power to analyze and interpret. Finally, 541 respondents with intermittent participation (i.e., respondents with missing interviews in the middle of their follow-up period) were excluded because of the modeling strategy. The final analytic sample consisted of 8,331 respondents.

### Variables

#### Dependent variable: Chronic disease burden

The main outcome variable in this study was the count of chronic diseases (range: 0–7). We included all available self-reported chronic somatic diseases queried in the HRS data, each prompted by “Has a doctor ever told you that you have…”: heart disease (including myocardial infarction, coronary heart disease, angina, congestive heart failure, or other heart problems), hypertension, stroke (excluding transient ischemic attack), diabetes, arthritis, lung disease (including chronic bronchitis or emphysema and excluding asthma), and cancer (including any malignant tumors with the exception of skin cancer).

We draw from the conceptual framework of multimorbidity that highlights the persistent, incurable, and chronic nature of disease accumulation and postulates that once a respondent has developed a disease, the respondent continues to have the disease even if the disease symptoms are attenuated through lifestyle changes or medication [22]. Clinically-inconsistencies disease response patterns exist in the self-report of chronic diseases across HRS waves (i.e., affirmative followed by negative responses) and are contrary to this conceptual framework. In order to address these inconsistencies, we applied a previously developed multistep adjudication method to our study sample’s responses [21]; briefly, discrepancies were resolved using disease-specific follow-up questions, which we considered indicative of disease status. Respondents with unresolved inconsistencies of conflicting chronic disease reports were excluded from the analysis (n = 844, 8.3%).

#### Key independent variable: Race/ethnicity

Race/ethnicity was defined using the two following questions: 1) “Do you consider yourself Hispanic or Latino?” and 2) “Do you consider yourself primarily white or Caucasian, Black or African American, American Indian, or Asian, or something else?” Three mutually-exclusive groups were used in the analyses: non-Hispanic white (hereafter white), non-Hispanic black (hereafter black), and Hispanic.

#### Covariates

Sociodemographic covariates included gender (male/female) and education (number of school years completed). Body-mass index (BMI) was calculated according to the established formula (BMI = weight [pounds] x 703 / height^2 [inches]) using respondents’ first self-reported height (HRS does not record height at each interview) and self-reported weight at each interview.

### Statistical analysis

Full and complete details of our methodological procedures are provided in the **S1 Appendix**. All analyses were conducted in STATA/SE 15 (StataCorp, College Station, TX), while figures were generated in RStudio version 1.1.456. The HRS over-samples black and Hispanic Americans, as well as older residents from Florida. Thus, we present weighted analyses to account for the oversampling as recommended by the HRS study [23]. Briefly, descriptive methods were used to summarize characteristics of our study population; frequencies and percentages were calculated for categorical variables while means and standard deviations or medians and interquartile ranges (IQR) were calculated for continuous variables. In order to assess the relationship between chronic disease accumulation and race/ethnicity, we estimated a series of negative binomial generalized estimating equation (GEE) models with a first-order autoregressive covariance structure. These models allowed us to estimate the accumulation of chronic diseases as individuals age, while accounting for the non-independence of observations for an individual over time. The negative binomial regression was chosen to account for over-dispersion (most respondents had no chronic conditions at baseline), assessed with Pearson’s chi-squared dispersion statistic, and chosen over alternate models based on fit assessed by comparing Bayesian Information Criterion (BIC) values [24,25]. We explored linear, quadratic, and cubic time terms in all models and determined the linear specification resulted in the best fit. Variance adjustments for the complex sampling design are not available for negative binomial GEE analyses in STATA/SE 15.

To control for the influence of loss to follow-up due to mortality and other attrition, we estimated models weighted by inverse probability weights (IPW) for missingness [26]. We account for differential loss to follow-up because of extensively documented racial and ethnic disparities in mortality [27,28]. Additional analyses comparing the inclusion and exclusion of decedents (not shown) demonstrated equivalent findings and provided support for the use of trimmed IPW weights to account for missingness regardless of the specific reason for loss to follow-up (i.e., death or attrition). Our final logistic model for missingness included chronic disease diagnoses, sociodemographic covariates, lifestyle, healthcare utilization, and health status variables (full list of model predictors in **Tables J-L in S1 Appendix**). We subsequently trimmed the IPWs to achieve a more balanced distribution of the weights [29], where respondents with IPWs ≥98^th^ percentile of the distribution were removed from the analysis (n = 141 or 0.01% of the sample; **Table Q in S1 Appendix** compares trimmed and untrimmed models to guide decision making for this approach).

Model 0 comprised the unconditional GEE model and reports the averaged chronic disease accumulation for all respondents over time. Model 1 comprised the base GEE model weighted by trimmed IPWs, and included coefficients for race/ethnicity, time and a race/ethnicity by time interaction. The interaction term tests for differences in accumulation of chronic diseases (difference in slope) according to race/ethnicity. Model 2 represents the covariate-adjusted GEE model weighted by trimmed IPWs. Covariates included time-invariant sociodemographic factors (race/ethnicity, education, female gender), while BMI was treated as time-varying.

We present the unexponentiated model coefficients (log odds), the exponentiated model coefficients or incident rate ratios (IRR), and the predicted values of chronic disease from model post-estimation commands (Stata procedure *margins* and *lincom*, respectively). The post-estimation predicted values reflect the marginal effect of race/ethnicity on the baseline count of chronic conditions and the estimated effect of time on the accumulation of chronic disease calculated at the mean value of covariates, where applicable. Finally, we conducted sensitivity analyses to explore the potential contribution of including net worth (i.e., income and assets after accounting for outstanding debts) to the covariate-adjusted model and tested three-way interactions between time, race/ethnicity, and BMI as well as between time, race/ethnicity, and gender.

## Results

The analytic study sample (**Table 1**) consisted of 8,331 participants, with a mean age at first interview of 53 years (SD = 1.4) and a mean age at last interview of 61 years (SD = 5.1). Fifty-seven percent of participants were female, 64% were white, 21% were black, and 15% were Hispanic. **Table 2** presents the negative binomial GEE models that specify baseline levels (intercept) and changes in chronic disease accumulation (slope) over the study period. Model 0 specifies a positive slope, indicating an increase in chronic disease accumulation over time for all study respondents (IRR 1.139, 95% CI, 1.35, 1.143). Model 1 reports the race/ethnicity adjusted model and indicates black respondents have initial chronic disease counts that are approximately 40% higher than white respondents (IRR 1.395, 95% CI 1.335, 1.443). With respect to the slope, black respondents have rates of accumulation that are approximately 1.3% slower than white respondents (IRR 0.987, 95% CI 0.978, 0.995) and Hispanic respondents have rates of chronic disease accumulation that are approximately 1.6% faster than white respondents (IRR 1.016, 95% CI 1.004, 1.029). Model 2 presents the covariate-adjusted results, and indicates black respondents have baseline chronic disease counts that are approximately 28% higher than white respondents (IRR 1.279, 95% CI 1.201, 1.361), Hispanic respondents have baseline chronic disease counts that are approximately15% lower than white respondents (IRR 0.852, 95% CI 0.775, 0.938). Black respondents have rates of chronic disease accumulation that are approximately 1.1% slower than white respondents (IRR 0.989, 95% CI 0.981, 0.998) and Hispanic respondents have rates of chronic disease accumulation that are approximately 1.5% faster than white respondents (IRR 1.015, 95% CI 1.002, 1.028). In addition, greater educational attainment is significantly associated with slower accumulations of chronic disease (IRR 0.956, 95% CI 0.938, 0.953), women have significantly greater accumulations of chronic disease relative to men (IRR 1.047, 95% CI 1.00, 1.092), and higher BMI is significantly associated with greater accumulations of chronic disease (IRR 1.011, 95% CI 1.009, 1.013).

**Table 1:** Descriptive characteristics of the study population, HRS 1998–2014.

Characteristic	Overall	Non-Hispanic White	Non-Hispanic Black	Hispanic
N	8331 (100)	5341 (64.1%)	1775 (21.3%)	1215 (14.6%)
Ever proxy interview	299 (3.6%)	208 (3.9%)	45 (2.5%)	46 (3.8%)
Age at first interview, mean (SD)	53.0 (1.4)	53.0 (1.4)	53.0 (1.4)	53.1 (1.4)
Age at last interview, mean (SD)	61.0 (5.1)	61.7 (5.1)	59.8 (4.8)	59.7 (4.5)
Gender, female	4759 (57.1%)	2981 (55.8%)	1104 (62.2%)	674 (55.5%)
Education level, median (IQR)	13 (12, 16)	14 (12, 16)	12 (12, 14)	12 (6, 14)
BMI at 1st interview, mean (SD)	29.0 (6.3)	28.3 (6.0)	30.9 (7.1)	29.5 (6.0)
Count of conditions at 1st interview, mean (SD)	1.06 (1.10)	0.98 (1.05)	1.37 (1.20)	1.00 (1.08)
Number of interviews, mean (SD)	4.9 (2.4)	5.3 (2.4)	4.4 (2.2)	4.3 (2.0)
Follow-up				
Deceased	511 (6.1%)	323 (6.1%)	136 (7.7%)	52 (4.3%)
Loss to follow-up	1476 (17.8%)	972 (18.2%)	285 (16.1%)	219 (18.0%)

**Table 2:** Negative binomial GEE models of chronic disease accumulation over time, HRS 1998–2014^1^^-^^2^.

		Model 0^3^		Model 1^4^		Model 2^5^	
		β (95% CI)	IRR (95% CI)	β (95% CI)	IRR (95% CI)	β (95% CI)	IRR (95% CI)
Baseline count of chronic diseases^6^^-^^7^	Overall	0.026 (-0.000, 0.052)	1.026 (1.000, 1.053)	-	-	-	-
	White	-	-	ref	ref	ref	ref
	Black	**-**	**-**	**0.333 (0.270, 0.396)**	**1.395 (1.310, 1.485)**	**0.246 (0.184, 0.308)**	**1.279 (1.201, 1.361)**
	Hispanic	-	-	0.012 (-0.074, 0.097)	1.012 (0.929, 1.101)	**-0.160 (-0.255, -0.064)**	**0.852 (0.775, 0.938)**
Accumulation of chronic diseases^8^	Time	**0.130 (0.127, 0.134)**	**1.139 (1.35, 1.143)**	**-**	-	-	-
	White*Time	-	-	ref	ref	ref	ref
	Black*Time	-	**-**	**-0.013 (-0.022, -0.005)**	**0.987 (0.978, 0.995)**	**-0.011 (-0.020, -0.002)**	**0.989 (0.981, 0.998)**
	Hispanic* Time	**-**	**-**	**0.016 (0.004, 0.029)**	**1.016 (1.004, 1.029)**	**0.015 (0.002, 0.027)**	**1.015 (1.002, 1.028)**
Covariates	Education	-	-	**-**	**-**	**-0.056 (-0.064, -0.048)**	**0.956 (0.938, 0.953)**
	Female	-	-	-	-	**0.046 (0.003, 0.088)**	**1.047 (1.00, 1.092)**
	BMI	-	-	**-**	**-**	**0.011 (0.009, 0.013)**	**1.011 (1.009, 1.013)**

Abbreviations: GEE = generalized estimating equations; HRS = Health and Retirement Study; IRR = incidence rate ratios; CI = confidence interval; BMI = body mass index.

^1^ Models were constructed to compare Non-Hispanic Black and Hispanic respondents to Non-Hispanic White respondents (reference group); **bolded** estimates are statistically significant at the p<0.05 alpha-level.

^2^ Results are presented as estimated model coefficients (β) and exponentiated β-coefficients or incident rate ratios (IRR).

^3^ Model 0—unconditional GEE model.

^4^ Model 1—GEE model weighted by IPW*baseline HRS respondent weights.

^5^ Model 2—GEE model weighted by IPW*baseline HRS respondent weights and adjusted for education, gender, and time-varying BMI.

^6^ Baseline count of chronic diseases (intercept), Model 0 interpretation: β-coefficient is the difference in log(count) of chronic conditions; IRR is the proportional difference in baseline chronic disease count relative to the counterfactual.

^7^ Baseline count of chronic diseases (intercept), Models 1 & 2 interpretation: β-coefficients are the difference in log(count) of chronic conditions relative to Non-Hispanic white respondents at baseline; IRRs are the proportional difference in baseline chronic disease count relative to Non-Hispanic white respondents and can be interpreted as “Non-Hispanic black respondents had a baseline count of chronic diseases 1.395 times higher than Non-Hispanic white respondents.”

^8^ Accumulation of chronic diseases (slope): IRR is the proportional difference in accumulation of chronic disease relative to Non-Hispanic white respondents. This can be interpreted as “Non-Hispanic black respondents accumulate chronic disease at a rate 0.987 times slower than Non-Hispanic white respondents.”

**Table 3** presents the post-estimation predicted values of chronic disease across models and reported at the first time period, or ages 51–55, and at the last time period, or ages 69–71 (predicted values across all time periods reported in **Table S in S1 Appendix**). On average, study participants start with 1.03 chronic diseases and end with 2.91 chronic diseases according to the unadjusted model (Model 0). According to Model 2 estimates, white respondents start with 0.98 chronic diseases and end with 2.8 chronic diseases; black respondents start with 1.3 chronic diseases and end with 3.3 chronic diseases; and Hispanic respondents start with 0.84 chronic diseases and end with 2.7 chronic diseases.

**Table 3:** Predicted values for chronic disease accumulation over time, HRS 1998–2014^1^^,^^2^.

	Model 0	Model 1			Model 2		
Time period	Overall	Non-Hispanic White	Non-Hispanic Black	Hispanic	Non-Hispanic White	Non-Hispanic Black	Hispanic
First (age 51–55)	1.026 (0.014)	0.982 (0.016)	1.370 (0.038)	0.994 (0.040)	0.983 (0.016)	1.258 (0.034)	0.838 (0.038)
Last (age 69–71)	2.908 (0.032)	2.797 (0.037)	3.509 (0.082)	3.223 (0.109)	2.811 (0.038)	3.295 (0.083)	2.695 (0.105)

Abbreviations: HRS = Health and Retirement Study.

^1^Results are presented as predicted values derived from post-estimation marginal effects for the baseline count of chronic conditions and linear combinations of coefficients for the accumulation of chronic diseases calculated at the mean of covariate values in the covariate-adjusted model.

^2^Standard errors reported in parentheses.

**Fig 1:**
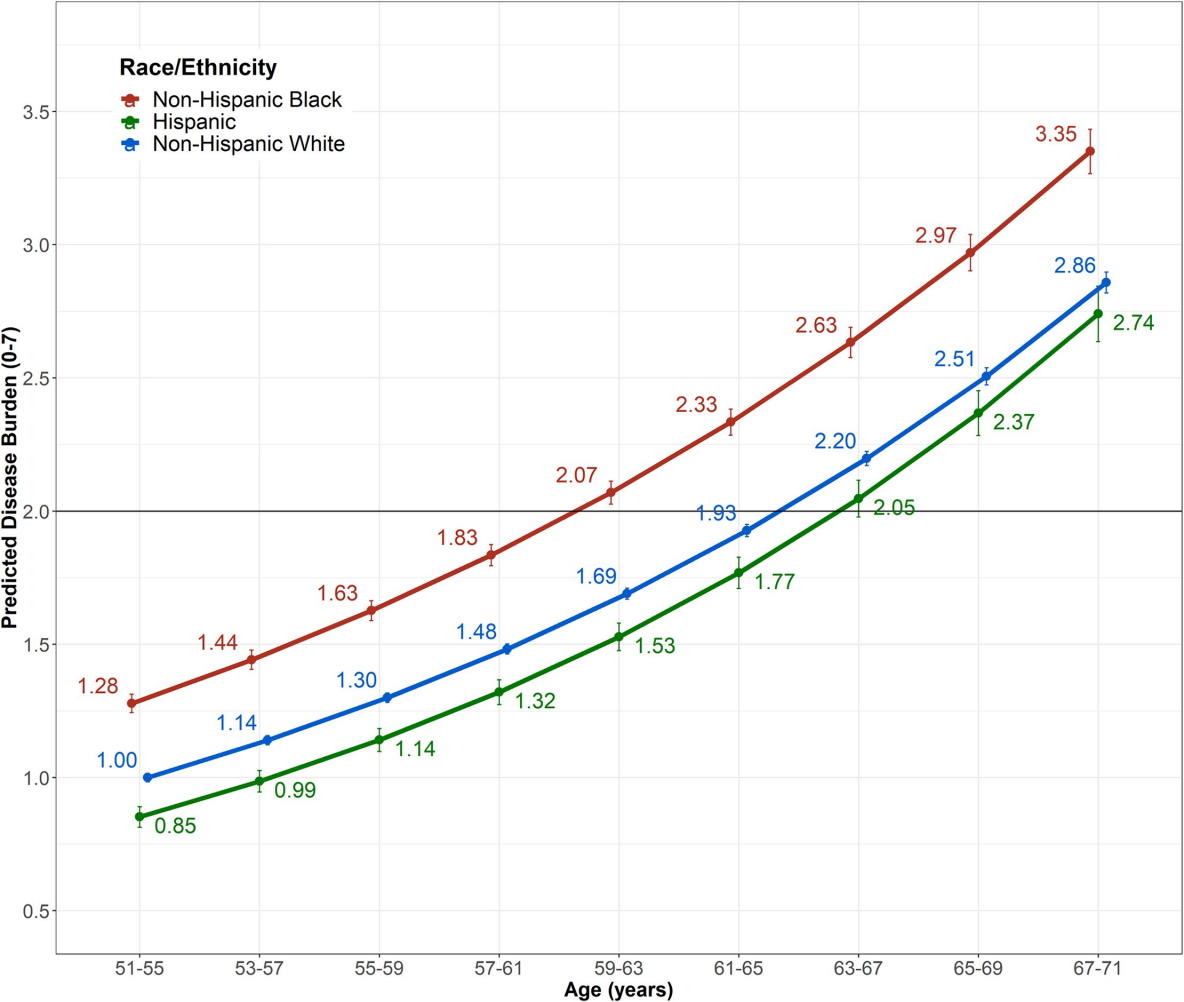
Non-Hispanic black, Non-Hispanic white, and Hispanic trajectories of chronic disease accumulation over time, HRS 1998–2014.

**Fig 1** graphically presents findings from the covariate-adjusted model (Model 3) to visualize the trajectories of chronic disease accumulation for white, black, and Hispanic respondents. The horizontal line represents the multimorbidity threshold of having two or more chronic diseases. Black respondents had significantly higher baseline levels of chronic disease compared to white respondents and exhibit a decreasing rate of chronic disease accumulation relative to white respondents. In addition, on average, black respondents cross the multimorbidity threshold approximately four years earlier than white respondents. In contrast, initial levels of chronic disease were significantly lower for Hispanic relative to white respondents, but the rate of chronic disease accumulation is significantly greater for Hispanic relative to white respondents over the study period.

In sensitivity analyses, the addition of net worth into the covariate-adjusted model did not result in statistically significant findings for net worth nor did this addition change the estimates of other models parameters. Finally, in testing three-way interactions between time, race/ethnicity, and BMI as well as between time, race/ethnicity, and gender across all model specifications, none of these interactions were statistically significant. Sensitivity analyses results are not presented but are available upon request.

## Discussion

This study assessed the accumulation of chronic disease and traced the progression of multimorbidity over a 16-year period among a middle-aged nationally-representative population in the United States. We sought to specify chronic disease accumulation and multimorbidity progression at the earliest age we observe HRS respondents—from their early to mid-50s to the most recent finalized data collection. As a result, our study adds to the multimorbidity literature by specifying changes in chronic disease accumulation and tracking differences in multimorbidity development for a diverse group of adults starting in middle-age. Several aspects of our findings are noteworthy. While it is unsurprising that middle-aged adults in the U.S. are accumulating chronic disease over time, black middle-aged adults had significantly higher initial levels of chronic disease burden compared with white counterparts. On average, black middle-aged adults crossed the multimorbidity threshold approximately four years prior to white middle-aged adults—mid-to-late 50s compared to early-to-mid 60s. Notably, Hispanic middle-aged adults accumulate disease at a faster rate than white middle-aged study participants. In combination, these findings suggest differential opportunities for preventing and reducing racial/ethnic disparities in multimorbidity—prior to entering middle age to delay the onset and lower the initial burden of chronic diseases among black adults, while focusing on preventing or slowing the accumulation of diseases in older age (late 60s and 70s) in the Hispanic population.

Our study complements findings from several prior studies [30–32] by addressing important gaps in our knowledge regarding the longitudinal development and progression of multimorbidity [33]. Similar to our findings, a study of multimorbidity incidence in a defined geographic region in the United States found that black adults were at greater risk of high disease burden and earlier multimorbidity onset [31]. However, in contrast to our prior study that examined racial and ethnic differences in chronic disease trajectories and found near-identical time trends between white and Mexican Americans [6], we found differences in the rate of change in chronic disease accumulation among Hispanic Americans. It is likely we observe a more rapid accumulation of chronic disease among Hispanics because our current study focuses on adults starting in their early to mid-50s, a susceptible period for age-related chronic diseases such as metabolic conditions that are more prevalent among Hispanic populations [34,35]. Our results highlight the importance of estimating multimorbidity trajectories starting in mid-life and make a case for preventing age-related chronic disease earlier in the lifecourse for at-risk populations.

Earlier lifecourse development of chronic disease and multimorbidity among non-Hispanic black Americans may indicate earlier and longer cumulative exposure to risk factors common to many of the chronic diseases that comprise multimorbidity. Risk factors that assault multiple body systems are prevalent among racial and ethnic minority populations in the U.S.—for example, obesity, chronically elevated stress levels, or elevated systemic inflammatory markers—and may, in turn, lead to earlier multimorbidity development [36–38]. In addition, poor access to good quality health care and low socioeconomic status may exacerbate and accelerate additional chronic disease development [39]. For example, Hispanic adults have greater prevalence of obesity and greater risk of developing diabetes in midlife [34]. If left uncontrolled or poorly controlled, these populations may be at risk of developing harbinger conditions—such as hypertension—that may be precursors for resulting complications, events, or additional diagnoses—such as myocardial infarction and stroke. A cascade of chronic disease following an influential diagnosis may account for the more rapid accumulation of chronic disease observed among Hispanic groups in this study.

Further, several publications across a range of international settings largely agree with our main results, finding greater accumulation of chronic disease and multimorbidity with older age and among groups with particular social or behavioral vulnerabilities [30–32]. A clinical study sample in the Netherlands reported that a small group (~5%) of patients seen in family practice clinics account for a large (>20%) proportion of reported chronic diseases, due to “intensive” multimorbidity patterns of 10 or more diseases accumulated over the lifecourse, and called for identifying factors associated with increased vulnerability in this group [30]. Similarly, a study examining multimorbidity trends in a nationally-representative sample of Canadians reported earlier onset of multimorbidity in mid-life in recent cohorts and among obese adults and among low-income groups [32]. It is notable that our study found no association with wealth in either the level or rate of chronic disease accumulation, likely due to high correlation between race/ethnicity, education, and wealth in the United States. Future studies may need to investigate cohort differences in multimorbidity development across racial/ethnic groups, to support the development of initiatives and programs to address forthcoming healthcare needs for changing demographic trends among vulnerable groups.

This study has several strengths. First, the HRS provides ongoing, robust longitudinal data reflecting a wide array of health, sociodemographic, and patient-reported outcomes generalizable to the U.S. population of middle-aged and older adults. These data provide the opportunity to observe changes in participant reports of chronic disease burden and multimorbidity onset during critical periods of middle to late adulthood in a large, ongoing prospective cohort study. Second, this study contributes to the emerging literature on the epidemiology of multimorbidity by specifying the longitudinal progression of multimorbidity burden over 16 years. Third, the prospective design and oversampling of racial and ethnic minority populations in the HRS permits assessment of time-sequencing of chronic disease burden and accumulation, and enables the estimation of multimorbidity onset and progression between adults from various racial and ethnic backgrounds. Fourth, by accounting for differences in mortality and loss to follow-up, our study provides important insights into differential rates of chronic disease accumulation between racial and ethnic groups, an improvement over prior work. In these ways, our study contributes to the evolving literature on multimorbidity and provides key insights into the examination of multimorbidity from a longitudinal perspective.

We also note several limitations to this study. First, we used a limited number of chronic diseases available in the public-use data files of the HRS. Future steps include leveraging the Medicare-HRS data linkage to ascertain a greater number of diagnoses, specifically, the 20 chronic diseases recommended by the U.S. Department of Health and Human Services for the operationalization of multimorbidity: diseases associated with aging that are persistent, prevalent, and incurable [9,22]. Second, chronic disease diagnoses were self-reported, which may reflect underreporting or underdiagnosis of chronic disease, particularly for adults with low health literacy, or those who experience lack of access to a usual source of care. Still, for adults with healthcare access, several studies have shown adequate concordance between patient reports of various physician-diagnosed chronic diseases and administrative or clinical data sources [40–42], and many studies highlight the importance of documenting patient reported outcomes of disease status [43–45]. Third, because of the limited number of American Indians, Asians and those who identified as “other” we did not have sufficient power to include them in the models. These are vulnerable population which should be studied. Fourth, these observational survey data provide associations and do not imply causation. Finally, standard error adjustments were not available for the analyses conducted, however small p-values suggest there would be few differences in the overall conclusions had adjustments been possible.

While our study centers on quantifying and identifying trends in chronic disease accumulation and multimorbidity development over time, we see these examinations as a first step in a progression of important research questions. We were unable to ascertain the severity of chronic disease and multimorbidity burdens with these data. Future research is needed to investigate the role of clinical presentation and severity of component diseases or prognosis, as well as the association between multimorbidity progression and outcomes important for older adults, such as functional disability, dementia, frailty, and other geriatric syndromes. Further, we did not examine preventive healthcare services that may be related to accumulation of chronic conditions. Lastly, our study did not focus on the mechanisms or underlying reasons for observed differences in multimorbidity and chronic disease accumulation. Further research is needed to more carefully examine the joint processes of morbidity and mortality among racially diverse middle and older adults, and to understand whether the higher rate of accumulation for Hispanics in late mid-life was related to more global health differences or access to Medicare benefits and improved access to healthcare that may address issues of underdiagnosis earlier in the lifecourse for this population.

Our study has important clinical and research implications by suggesting that multimorbidity is not only common, but emerging earlier in middle age and progressing more rapidly for underrepresented racial and ethnic groups in the United States. Our findings raise significant public health and healthcare system challenges for how best to avert and delay further compounding of clinical complexity among older adults. Specifically, these results highlight the need for proactive interventions and policies designed to optimize clinical care, preserve functional independence, and delay institutionalization for patients with high multimorbidity burden, particularly those from minority racial and ethnic backgrounds.

## Supporting information

S1 Appendix Comprehensive analysis documentation. (DOCX)
