# Supplementary material for: Racial/ethnic differences in multimorbidity development and chronic disease accumulation for middle-aged adults
Source: PLoS One. 2019 Jun 17;14(6):e0218462. doi: 10.1371/journal.pone.0218462 (PMC6576751; doi:10.1371/journal.pone.0218462)
Supplement: S1 Appendix — (DOCX) [file pone.0218462.s001.docx]

**S1 Appendix. Comprehensive Analysis Documentation**

**Decisions**

- Scope of this analysis is on somatic disease. Include the following chronic conditions in count: arthritis, cancer, diabetes, heart disease, hypertension, lung disease, and stroke. We did not include cognitive impairment and depression - the way cognitive impairment and depression are measured (cognitive performance and depressive symptoms) is not consistent with the doctor-diagnosed manner in which somatic information is collected. How we are conceptualizing disease accumulation (monotonically increasing) is not consistent with cognitive and mental conditions are queried as it is possible for a respondent to recover from symptoms and improve performance.
- Inclusion/Exclusion:
  - Exclude respondents with disease response patterns that are clinically inconsistent with persistent and incurable chronic diseases, and not consistent with the conceptualization of chronic disease accumulation (Yes to No response pattern). We provide demographic information on these respondents.
  - Remove ‘Other’ race/ethnicity group; we do not have sufficient power to detect differences in effect of time according to race/ethnicity if ‘Other’ group is further refined, e.g. if we use race/ethnicity categories of Pacific Islander, Asian, American Indian, etc.
  - Exclude respondents with intermittent participation in study - this is done as a product of GEE modeling. When there is intermittent participation it is not possible to estimate an autoregressive correlation structure, and the respondent is excluded from estimation. Still, we are able to estimate an autoregressive correlation if missingness occurs at the end of a respondent’s follow-up period, so we are able to include decedents and those permanently lost to follow-up.
  - Exclude respondents with one interview only - this is done as a product of GEE modeling
- Analysis plan:
  - Model outcome (count of chronic conditions) using a negative binomial model; model fit statistics indicate that a negative binomial model will fit better than a Poisson regression
  - Use a GEE model to account for the within respondent correlation of measures and will use a first-order autoregressive correlation structure (AR1)
  - Incorporate HRS complex survey design into analysis
    - Account for sampling probability by weighting model with respondent’s HRS weight at their first interview (e.g. baseline survey weight)
  - Treatment of covariates:
  - Education will be continuous (years of education) BMI will be time-varying Level of statistical significance - alpha=0.01
  - Inverse probability of participation (non-participation=death and attrition)
    - Account for HRS complex survey design in logistic model
    - Compare models with and without trimming high IPW values
- Additional analysis options explored:
  - Explore the inclusion of net worth in covariate adjusted models as a time-varying covariate; was not statistically significant and did not change other model parameters
  - Explore the addition of quadratic or cubic time terms in all models; higher order terms were all non-significant
  - Explore interactions between time & BMI (continuous and categorical) & race/ethnicity, and time & education & race/ethnicity; none of these interactions were statistically significant
  - We explored the 3-way interaction between time by *BMI by* race/ethnicity with BMI being a 4-category variable (not significant; data not shown and can be presented upon request) as well as a 3-way interaction between time by gender by race/ethnicity (not significant; data not shown and can be presented upon request).

**Sample Size**

There are a total of 38,183 respondents in the Health & Retirement Study. Over the period from 1998-2014, there were 10,126 respondents who participated in at least one cohort-eligible interview and who were aged 51-55 for one of these interviews. Of these respondents, 8,872 had questionnaire responses that were consistent over time (all No to Yes answers or No to No answers); were not missing the primary outcome at interview 1 (primary outcome=number of conditions); were not missing race/ethnicity information; and, identified as either Non-Hispanic White, Non-Hispanic Black, or Hispanic. Due to our modeling strategy, respondents with inconsistent participation patterns and only one qualifying interview were excluded. Thus, our sample size for modeling purposes was 8,331 respondents.


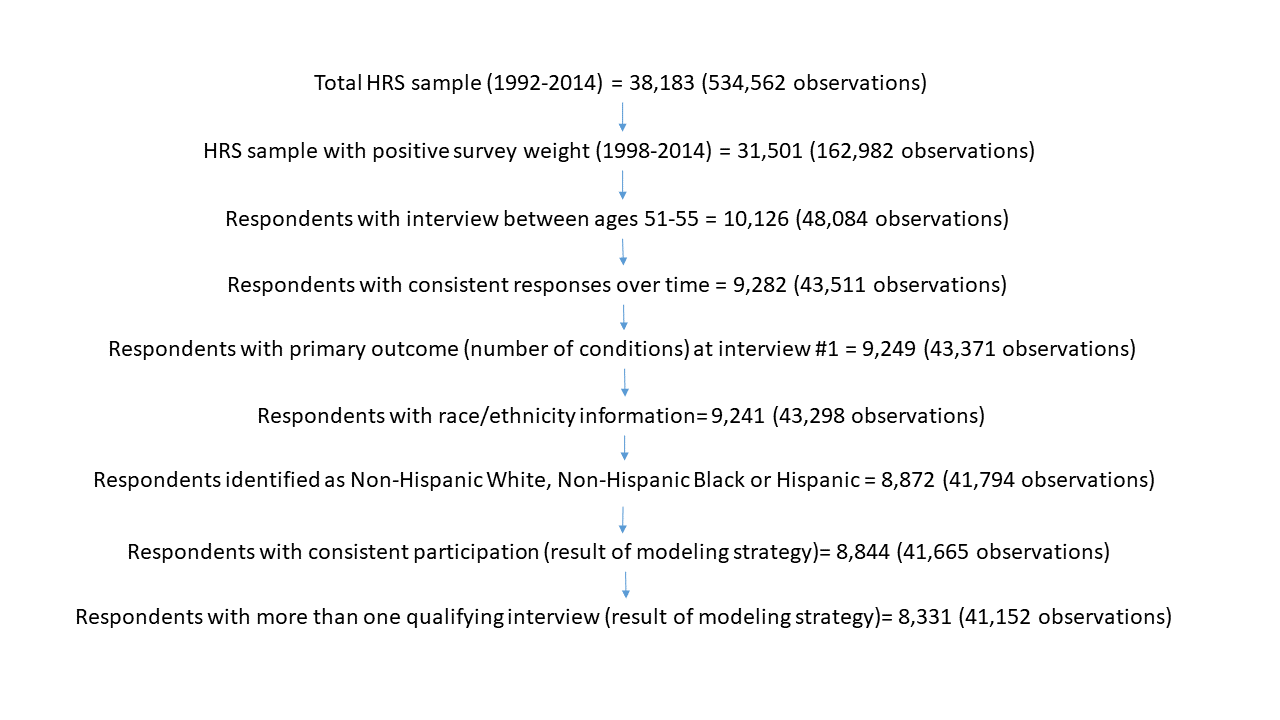


**S1 Figure A**. Sample Size Flow Chart

**S1 Table A.** Demographic characteristics of study population

. table1 if table==1 & sample1==1, by(race_ethnicity) vars(p bin \ female bin \ ed conts \ bmi contn \ conditionsr conts) format(%2.1f)
 +------------------------------------------------------------------------------------------------------------------+
 | Factor Non-Hispanic White Non-Hispanic Black Hispanic p-value |
 |------------------------------------------------------------------------------------------------------------------|
 | N 5341 1775 1215 |
 |------------------------------------------------------------------------------------------------------------------|
 | Ever proxy interview 402 (7.5%) 88 (5.0%) 102 (8.4%) <0.001 |
 |------------------------------------------------------------------------------------------------------------------|
 | Gender, female 2981 (55.8%) 1104 (62.2%) 674 (55.5%) <0.001 |
 |------------------------------------------------------------------------------------------------------------------|
 | Education level, median (IQR) 14.0 (12.0, 16.0) 12.0 (12.0, 14.0) 12.0 (6.0, 14.0) <0.001 |
 |------------------------------------------------------------------------------------------------------------------|
 | BMI at 1st interview, mean (SD) 28.3 (6.0) 30.9 (7.1) 29.5 (6.0) <0.001 |
 |------------------------------------------------------------------------------------------------------------------|
 | Conditions at 1st interview, median (IQR) 1.0 (0.0, 2.0) 1.0 (1.0, 2.0) 1.0 (0.0, 2.0) <0.001 |
 +------------------------------------------------------------------------------------------------------------------+

**S1 Table B.** Demographic characteristics of n=844 respondents excluded due to unresolved inconsistencies in disease responses

. table1 if branchpoint==3 & ytn==1 & interview==0, ////vars(p bin \ age contn \ female bin \ race_ethnicity cat \ ed

conts \ bmi contn \ conditionsr conts) format(%2.1f)

 +------------------------------------------------------------------------------------+
 | Factor Level Value |
 |------------------------------------------------------------------------------------|
 | N 844 |
 |------------------------------------------------------------------------------------|
 | Ever proxy interview 153 (18.1%) |
 |------------------------------------------------------------------------------------|
 | Age, mean (SD) 53.1 (1.4) |
 |------------------------------------------------------------------------------------|
 | Gender, female 576 (68.2%) |
 |------------------------------------------------------------------------------------|
 | Race/Ethnicity Non-Hispanic White 539 (63.9%) |
 | Non-Hispanic Black 139 (16.5%) |
 | Hispanic 133 (15.8%) |
 | Other 33 (3.9%) |
 |------------------------------------------------------------------------------------|
 | Education level, median (IQR) 12.0 (12.0, 15.0) |
 |------------------------------------------------------------------------------------|
 | BMI at 1st interview, mean (SD) 29.2 (6.3) |
 |------------------------------------------------------------------------------------|
 | Conditions at 1st interview, median (IQR) 2.0 (1.0, 3.0) |
 +------------------------------------------------------------------------------------+

**Interviews, number of conditions and accumulation of conditions over time**

Average age over the entire study period is 58.0 (SD=4.75).

**S1 Table C.** Tabulation of respondents by wave and interview number

. tab interview wave if sample1==1, m

 | study year
 interview | 1998 2000 2002 2004 2006 2008 2010 2012 2014 | Total
 -----------+---------------------------------------------------------------------------------------------------+----------
 0 | 2,077 223 31 2,394 207 13 3,193 193 0 | 8,331
 1 | 0 1,990 280 40 2,279 271 58 3,122 286 | 8,326
 2 | 0 0 1,859 276 49 2,112 310 75 2,866 | 7,547
 3 | 0 0 0 1,753 275 53 1,964 307 90 | 4,442
 4 | 0 0 0 0 1,646 291 58 1,834 301 | 4,130
 5 | 0 0 0 0 0 1,561 293 62 1,713 | 3,629
 6 | 0 0 0 0 0 0 1,469 286 58 | 1,813
 7 | 0 0 0 0 0 0 0 1,381 278 | 1,659
 8 | 0 0 0 0 0 0 0 0 1,275 | 1,275
 -----------+---------------------------------------------------------------------------------------------------+----------
 Total | 2,077 2,213 2,170 4,463 4,456 4,301 7,345 7,260 6,867 | 41,152

**S1 Table D.** Tabulation of Respondent’s number of conditions by interview number

. tab interview conditions7r if sample1==1, m

 | conditions7r
 interview | 0 1 2 3 4 5 6 7 | Total
 -----------+----------------------------------------------------------------------------------------+----------
 0 | 3,113 2,810 1,548 578 201 71 9 1 | 8,331
 1 | 2,558 2,758 1,778 798 304 103 26 1 | 8,326
 2 | 1,912 2,405 1,800 912 352 128 35 3 | 7,547
 3 | 952 1,376 1,114 623 270 79 25 3 | 4,442
 4 | 736 1,190 1,091 677 306 99 26 5 | 4,130
 5 | 512 993 1,008 650 308 122 31 5 | 3,629
 6 | 198 421 516 397 194 65 20 2 | 1,813
 7 | 146 363 452 387 214 73 22 2 | 1,659
 8 | 91 241 352 308 202 59 18 4 | 1,275
 -----------+----------------------------------------------------------------------------------------+----------
 Total | 10,218 12,557 9,659 5,330 2,351 799 212 26 | 41,152

**S1 Table E.** Tabulation of Respondent’s change in number of conditions by interview number

. tab interview accumulate if sample1==1, m

 | accumulate
 interview | 0 1 2 3 4 5 . | Total
 -----------+-----------------------------------------------------------------------------+----------
 0 | 0 0 0 0 0 0 8,331 | 8,331
 1 | 6,598 1,226 158 23 2 0 319 | 8,326
 2 | 6,041 1,199 146 11 1 0 149 | 7,547
 3 | 3,576 656 82 7 1 1 119 | 4,442
 4 | 3,379 579 68 16 2 0 86 | 4,130
 5 | 2,992 543 52 4 0 0 38 | 3,629
 6 | 1,446 298 31 4 0 0 34 | 1,813
 7 | 1,379 243 16 3 0 0 18 | 1,659
 8 | 1,088 165 19 3 0 0 0 | 1,275
 -----------+-----------------------------------------------------------------------------+----------
 Total | 26,499 4,909 572 71 6 1 9,094 | 41,152

**Mean number of conditions over time, overall and by race/ethnicity**

**S1 Table F.** Mean number of conditions by interview number

| ***Inter*** | ***Conditions*** | ***Difference*** |
| --- | --- | --- |
| **1** | 1.06 ( 1.10) | . ( .) |
| **2** | 1.27 ( 1.20) | 0.20 ( 0.47) |
| **3** | 1.46 ( 1.26) | 0.21 ( 0.46) |
| **4** | 1.60 ( 1.29) | 0.20 ( 0.46) |
| **5** | 1.77 ( 1.33) | 0.19 ( 0.47) |
| **6** | 1.93 ( 1.35) | 0.18 ( 0.43) |
| **7** | 2.14 ( 1.35) | 0.21 ( 0.46) |
| **8** | 2.29 ( 1.37) | 0.17 ( 0.42) |
| **9** | 2.44 ( 1.37) | 0.17 ( 0.43) |
| **Total** | 1.52 ( 1.30) | 0.20 ( 0.46) |


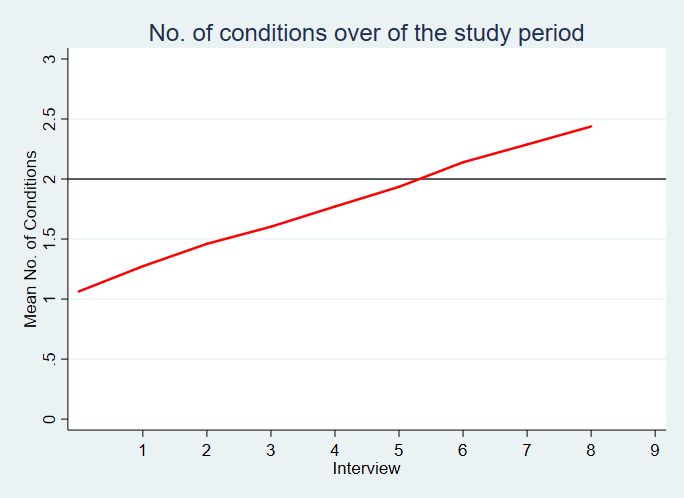


**S1 Figure B**. Number of conditions over the study period

**S1 Table G.** Mean number of conditions by interview number according to race/ethnicity

| ***Inter*** | ***NH White*** | ***NH Black*** | ***Hispanic*** |
| --- | --- | --- | --- |
| **1** | 0.98 ( 1.05) | 1.37 ( 1.20) | 1.00 ( 1.08) |
| **2** | 1.17 ( 1.16) | 1.62 ( 1.28) | 1.23 ( 1.17) |
| **3** | 1.35 ( 1.21) | 1.83 ( 1.33) | 1.45 ( 1.26) |
| **4** | 1.50 ( 1.24) | 2.03 ( 1.41) | 1.65 ( 1.30) |
| **5** | 1.67 ( 1.29) | 2.20 ( 1.40) | 1.83 ( 1.35) |
| **6** | 1.83 ( 1.31) | 2.36 ( 1.45) | 2.04 ( 1.33) |
| **7** | 2.05 ( 1.33) | 2.54 ( 1.39) | 2.20 ( 1.37) |
| **8** | 2.20 ( 1.35) | 2.73 ( 1.37) | 2.35 ( 1.38) |
| **9** | 2.34 ( 1.36) | 2.95 ( 1.37) | 2.51 ( 1.29) |
| **Total** | 1.45 ( 1.27) | 1.84 ( 1.38) | 1.45 ( 1.28) |


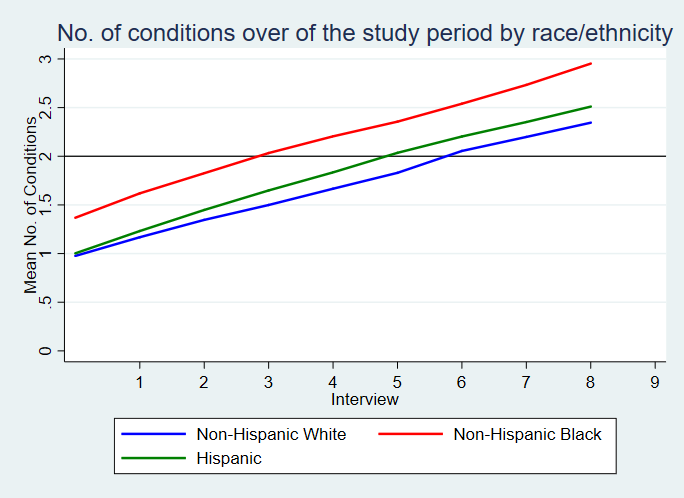


**S1 Figure C**. Number of conditions over the study period by race/ethnicity

**Models: Choice of Negative Binomial Model**. With regard to the use of negative binomial compared to other count models (Poisson, zero-inflated negative binomial, zero-inflated Poisson), the negative binomial model specification was chosen based on model fit statistics. At baseline, the best fitting model is the negative binomial according to Stata statistical software manual recommendations to use an information criterion to determine best fit.

|  | BIC | Test | p-value |
| --- | --- | --- | --- |
| Poisson | 22780.27 | - | - |
| Negative binominal | 22733.72 | LR test of alpha=0^1^ | <0.0001 |
| Zero-inflated negative binomial | 22748.25 | Voung test^2^ | 0.0344 |
| ^1^ Compares Poisson and negative binomial models | | | |
| ^2^ Compares negative binomial and zero-inflated negative binomial models.  **NOTE from Stata User Guide**: “Greene (1994) proposed using the Vuong test for nonnested models (Vuong 1989) to test for zero inflation. Despite many earlier citations, recent work by Wilson (2015) has shown that the Vuong test is inappropriate for testing zero inflation. Nesting occurs when the probability of zero inflation is 0, which is on the boundary, and this violates the regularity conditions of the Vuong test for nonnested models. So the distribution of the test statistic is not standard normal. The actual distribution is unknown and thus cannot be used for inference. You may consider using information criteria to choose between the standard and the zero-inflated models.” | | | |

**Models: Non-IPW negative binomial models**

Negative binomial models include:

- Model 1: Time (interview) only

- Model 2: Time and race/ethnicity

- Model 3: Time, race/ethnicity, time*race/ethnicity

All GEE negative binomial models adjust for the correlation in measures within a respondent using first-order autoregressive correlation structure. Using this correlation structure resulted in the elimination of 546 respondents who either participated intermittently (n=28) or who had only one interview (n=518); they were removed from the model as the correlation structure couldn’t be constructed given their response patterns (also reference to S1 Figure A). As well, GEE models are weighted by respondent’s entry year/baseline HRS respondent-level survey weight; this partially accounts for the complex survey design of HRS. S1 Table H compares models 1-3 and the text below will describe how to interpret the results. S1 Figure D plots the predicted values from models 3.

**NOTE:** We explored if the addition of a quadratic or cubic term for time (interview) would improve our model specification. In all models (non-IPW, IPW, covariate adjusted), the interaction between race/ethnicity and quadratic or cubic time was not statistically significant.

**S1 Table H.** Comparison of non-IPW NB models

. esttab model1 model2 model3, se mtitle label constant

 --------------------------------------------------------------------
 (1) (2) (3)
 model1 model2 model3
 --------------------------------------------------------------------
 interview 0.130*** 0.131*** 0.131***
 (0.00176) (0.00179) (0.00208)

 Non-Hispanic White 0 0
 (.) (.)

 Non-Hispanic Black 0.300*** 0.329***
 (0.0246) (0.0308)

 Hispanic 0.0594 0.0216
 (0.0326) (0.0423)

 Non-Hispanic White~w 0
 (.)

 Non-Hispanic Black~w -0.0116**
 (0.00426)

 Hispanic # interview 0.0147*
 (0.00610)

 Constant 0.0257 -0.0187 -0.0191
 (0.0133) (0.0152) (0.0159)
 --------------------------------------------------------------------
 Observations 41147 41147 41147
 --------------------------------------------------------------------
 Standard errors in parentheses
 * p<0.05, ** p<0.01, *** p<0.001

**Interpretation of model coefficients (S1 Table H)**

All coefficients represent the count of diseases on the log-scale. For instance, the constant/intercept in S1 Table H for model 1 is -.042 log(disease count), which translates to a population averaged disease count of 0.958 (e^-.042) over the course of the entire study period.

- Model 1:
  - Constant (Intercept): The log(disease count) when time is zero.
  - Interview (Slope): The increase in disease count over time on the log-scale. Can be translated into the % increase in disease count over time.
- Model 2:
  - Constant (Intercept): The log(disease count) for Non-Hispanic White respondents when time is zero.
  - Interview (Slope): The increase in disease count over time on the log-scale.
  - Non-Hispanic White (Explanatory factor): Reference group
  - Non-Hispanic Black (Explanatory factor): The difference in intercept between Non-Hispanic Black vs Non-Hispanic White respondents. We see that Non-Hispanic Black respondents had a higher log(disease count) at time zero when compared to Non-Hispanic White respondents (coefficient=0.306). This translates into a 35% higher disease count at time zero (e^0.306 = 1.35).
  - Hispanic (Explanatory factor): The difference in intercept between Hispanic vs Non-Hispanic White respondents.
- Model 3:
  - Constant (Intercept): The log(disease count) for Non-Hispanic White respondents when time is zero.
  - Interview (Slope): The increase in disease count for Non-Hispanic White respondents over time on the log-scale.
  - Non-Hispanic White (Explanatory factor): Reference group
  - Non-Hispanic Black (Explanatory factor): The difference in the intercept between Non-Hispanic Black vs Non-Hispanic White respondents.
  - Hispanic (Explanatory factor): The difference in the intercept between Hispanic vs Non-Hispanic White respondents.
  - Non-Hispanic White # Interview (Explanatory factor*Slope): Reference group
  - Non-Hispanic Black # Interview (Explanatory factor*Slope): The difference in slope between Non-Hispanic Black vs Non-Hispanic White respondents. We can translate these coefficients into a % increase in disease count over time of 12.4% for Non-Hispanic Black respondents (e^(0.128-0.0105)) and 13.6% for Non-Hispanic White respondents (e^0.128; NS difference).
  - Hispanic # Interview (Explanatory factor*Slope): The difference in slope between Hispanic vs Non-Hispanic White respondents.


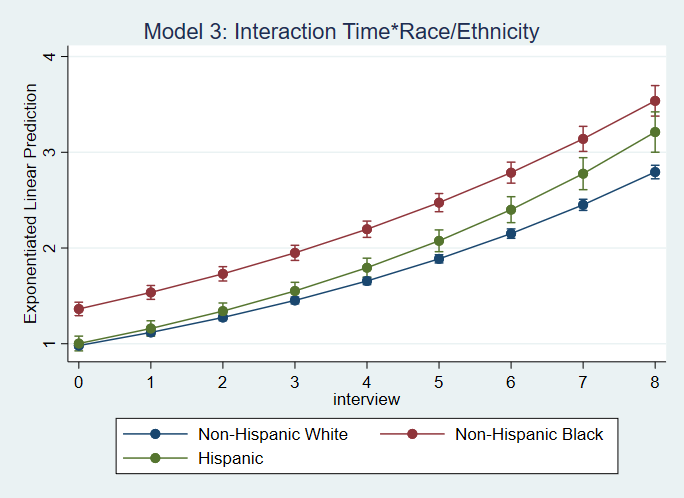


**S1 Figure D**. Model 3: Interaction of time by race/ethnicity

**Observed Data vs. Model 3 Predictions**

**S1 Table I.** Comparison of observed values and Model 3 linear prediction

| ***Iw*** | ***NH White*** |  | ***NH Black*** |  | ***Hispanic*** |  |
| --- | --- | --- | --- | --- | --- | --- |
| **1** | 0.98 ( 0.01) | 0.98 ( 0.02) | 1.37 ( 0.03) | 1.36 ( 0.04) | 1.00 ( 0.03) | 1.00 ( 0.04) |
| **2** | 1.17 ( 0.02) | 1.12 ( 0.02) | 1.62 ( 0.03) | 1.54 ( 0.04) | 1.23 ( 0.03) | 1.16 ( 0.04) |
| **3** | 1.35 ( 0.02) | 1.27 ( 0.02) | 1.83 ( 0.03) | 1.73 ( 0.04) | 1.45 ( 0.04) | 1.34 ( 0.04) |
| **4** | 1.50 ( 0.02) | 1.45 ( 0.02) | 2.03 ( 0.05) | 1.95 ( 0.04) | 1.65 ( 0.06) | 1.55 ( 0.05) |
| **5** | 1.67 ( 0.02) | 1.66 ( 0.02) | 2.20 ( 0.05) | 2.20 ( 0.04) | 1.83 ( 0.06) | 1.79 ( 0.05) |
| **6** | 1.83 ( 0.03) | 1.89 ( 0.02) | 2.36 ( 0.06) | 2.47 ( 0.05) | 2.04 ( 0.07) | 2.08 ( 0.06) |
| **7** | 2.05 ( 0.04) | 2.15 ( 0.03) | 2.54 ( 0.08) | 2.79 ( 0.06) | 2.20 ( 0.12) | 2.40 ( 0.07) |
| **8** | 2.20 ( 0.04) | 2.45 ( 0.03) | 2.73 ( 0.09) | 3.14 ( 0.07) | 2.35 ( 0.12) | 2.78 ( 0.09) |
| **9** | 2.34 ( 0.04) | 2.79 ( 0.04) | 2.95 ( 0.11) | 3.54 ( 0.08) | 2.51 ( 0.13) | 3.21 ( 0.11) |

Sample Size: NH White, n=5,341; NH Black, n=1,775; Hispanic, n=1,215

**Models: Creation of IPW model**

Logistic regression was used to create the inverse probability weights (IPW) of participating fully over the study period. Respondents were considered non-participating if they were deceased or were lost to follow-up once they met our project’s inclusion criteria. The goal of model building was to predict being participation while minimizing the number of respondents with a missing IPW and minimizing extreme IPW values. We will take the HRS complex survey design into account in this model, which includes utilizing respondent baseline survey weights, the standard error computation unit and strata information. S1 Table J tabulates the number of respondents who fully participated and who did not fully participate over the study period (deceased or attrited respondents).

Many of the predictors considered for inclusion into the logistic model were collected/updated at each interview (such as hypertension and IADL/ADL). These variables were redefined as 1) ‘ever reported’ if the predictor was binary or 2) median reported value, over the study period, if the predictor was ordinal or continuous (self-rated health, BMI, IADL/ADL and number of conditions).

S1 Table K lists the predictors considered for inclusion into the logistic model. Model selection was an iterative process in which a predictor was removed and remained removed in the additional models unless otherwise noted; please see S1 Table L for reason for variables exclusion and IPW descriptives of generated models. Figures E & F display the distribution of IPW values for the final logistic model, before and after trimming of large IPW values.

Our final logistic model is model 5 from S1 Table K; model output is displayed in S1 Table M. This model generated IPW values for 8,272 respondents (n=59 without predicted values) and had the smallest maximum IPW value of all models. If extreme values are trimmed (values >=98th percentile), 204 respondents will be removed from any the subsequent IPW analyses. Of those that would be trimmed from our analyses, S1 Table N tabulates their race/ethnicity.

**S1 Table J.** Enumeration of full participation

. distinct hhidpn if sample1==1 & participate==0

 -------------------------------
 | total distinct
 --------+----------------------
 hhidpn | 8466 1987
 -------------------------------


 . distinct hhidpn if sample1==1 & participate==1

 -------------------------------
 | total distinct
 --------+----------------------
 hhidpn | 32686 6344
 -------------------------------

**S1 Table K.** Potential predictors of fully participating

. mdesc participate usborn female race_ethnicity ed coupled_ipw smoke_ipw alcohol_ipw srh_ipw ///
 incontinent_ipw pain_ipw work_ipw hosp_ipw nrshm_ipw ouptsurg_ipw rx_ipw proxy_ipw ///
 bmi_ipw bp_ipw diab_ipw cancr_ipw lung_ipw hrt_ipw strk_ipw arth_ipw depression_ipw cogimp_ipw ///
 ADLIADL_ipw conditions_ipw if table==1 & sample1==1


 Variable | Missing Total Percent Missing
 ----------------+-----------------------------------------------
 participate | 0 8,331 0.00
 usborn | 3 8,331 0.04
 female | 0 8,331 0.00
 race_ethni~y | 0 8,331 0.00
 ed | 55 8,331 0.66
 coupled_ipw | 0 8,331 0.00
 smoke_ipw | 3 8,331 0.04
 alcohol_ipw | 0 8,331 0.00
 srh_ipw | 0 8,331 0.00
 incontinen~w | 0 8,331 0.00
 pain_ipw | 0 8,331 0.00
 work_ipw | 0 8,331 0.00
 hosp_ipw | 1 8,331 0.01
 nrshm_ipw | 1 8,331 0.01
 ouptsurg_ipw | 1 8,331 0.01
 rx_ipw | 1 8,331 0.01
 proxy_ipw | 0 8,331 0.00
 bmi_ipw | 44 8,331 0.53
 bp_ipw | 0 8,331 0.00
 diab_ipw | 0 8,331 0.00
 cancr_ipw | 0 8,331 0.00
 lung_ipw | 0 8,331 0.00
 hrt_ipw | 0 8,331 0.00
 strk_ipw | 0 8,331 0.00
 arth_ipw | 0 8,331 0.00
 depression~w | 82 8,331 0.98
 cogimp_ipw | 83 8,331 1.00
 ADLIADL_ipw | 0 8,331 0.00
 conditions~w | 0 8,331 0.00
 ----------------+-----------------------------------------------

**S1 Table L.** Logistic models for IPW generation: weights and model fit^1^

|  | **IPW** | | | | |
| --- | --- | --- | --- | --- | --- |
| **Models (total possible n=8,331)** | **minimum** | **median** | **maximum** | **95% per** | **99% per** |
| 1 - All covariate listed above (n=8,143) | 1.07 | 1.27 | 8.53 | 1.84 | 2.42 |
| 2 - Remove depression & cognitive impairment (missing values; n=8,225) | 1.07 | 1.27 | 6.11 | 1.85 | 2.46 |
| 3 - Removing median self-rated health (collinearity; n=8,225) | 1.07 | 1.27 | 5.6 | 1.83 | 2.36 |
| 4 - Removing US born (coefficient not statistically significant; n=8,228) | 1.08 | 1.27 | 5.4 | 1.84 | 2.35 |
| 5 - Removing median BMI (missing values; n=8,272) | 1.1 | 1.28 | 5.19 | 1.82 | 2.33 |

^1^This was an iterative process in which a predictor was removed and remained removed in additional models unless noted otherwise


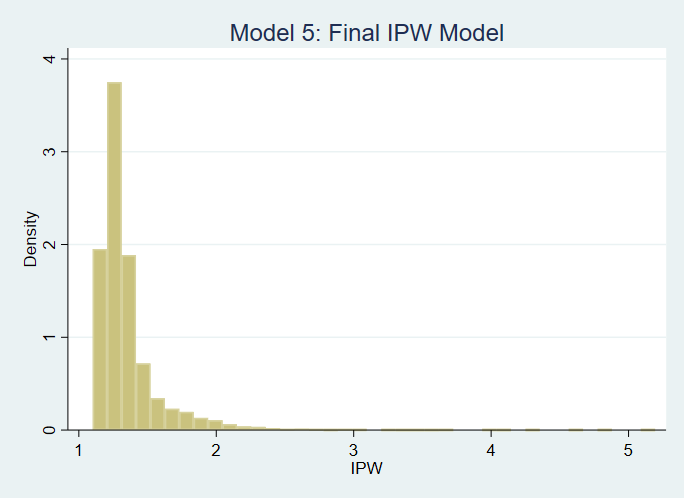


**S1 Figure E.** Model 5: Final IPW model


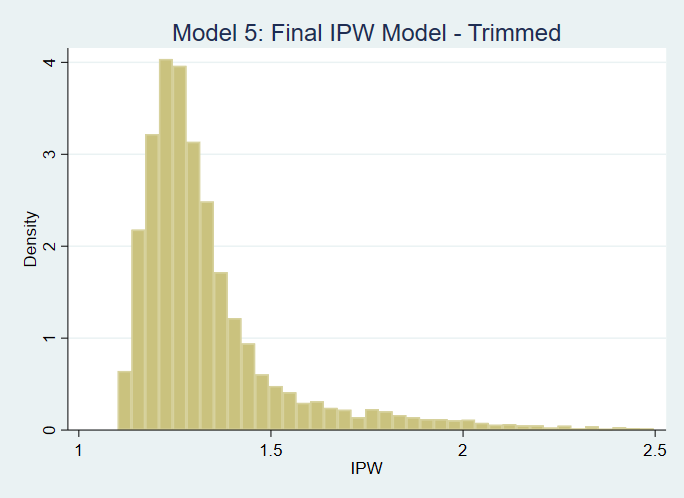


**S1 Figure F**. Model 5: Final IPW model, trimmed

**S1 Table M.** Output of final logistic model (Model 5)

. quietly svy linearized, subpop(if table==1 & sample1==1): ///
 logistic participate female race_ethnicity ed coupled_ipw smoke_ipw alcohol_ipw incontinent_ipw pain_ipw work_ipw hosp_ipw ///
 nrshm_ipw ouptsurg_ipw rx_ipw proxy_ipw bp_ipw diab_ipw cancr_ipw lung_ipw hrt_ipw strk_ipw arth_ipw ADLIADL_ipw conditions_ipw, or

**S1 Table N.** Trimmed respondents (IPW>=98th percentile): Tabulation of Race/Ethnicity

. tab race_ethnicity if table==1 & tile==1 & sample1==1

 Race/Ethnicity | Freq. Percent Cum.
 -------------------+-----------------------------------
 Non-Hispanic White | 118 57.84 57.84
 Non-Hispanic Black | 44 21.57 79.41
 Hispanic | 42 20.59 100.00
 -------------------+-----------------------------------
 Total | 204 100.00

**Correlation of Covariates**

**S1 Table O.** Correlation between socioeconomic covariates

. corr ed income networth black hispanic if sample1==1 & interviews==1
 (obs=8,256)

 | ed income networth black hispanic
 -------------+---------------------------------------------
 ed | 1.0000
 income | 0.3048 1.0000
 networth | 0.2130 0.5042 1.0000
 black | -0.0348 -0.1369 -0.1424 1.0000
 hispanic | -0.3774 -0.1231 -0.0870 -0.2137 1.0000


 . corr ed income networth black hispanic if sample1==1 & interviews==9
 (obs=1,275)

 | ed income networth black hispanic
 -------------+---------------------------------------------
 ed | 1.0000
 income | 0.3061 1.0000
 networth | 0.2813 0.5729 1.0000
 black | -0.1409 -0.1140 -0.1244 1.0000
 hispanic | -0.3397 -0.0658 -0.0580 -0.1124 1.0000

**S1 Table P.** Correlation between model covariates and race/ethnicity at baseline

. pbis black ed if sample1==1 & interview==0

 (obs= 8258)
 Np= 1762 p= 0.21
 Nq= 6496 q= 0.79
 ------------------+------------------+------------------+------------------+
 Coef.= -0.0351 t= -3.1937 P>|t| = 0.0014 df= 8256

 . pbis hispanic ed if sample1==1 & interview==0

 (obs= 8274)
 Np= 1208 p= 0.15
 Nq= 7066 q= 0.85
 ------------------+------------------+------------------+------------------+
 Coef.= -0.3840 t= -37.8268 P>|t| = 0.0001 df= 8272

 . pbis black bmi if sample1==1 & interview==0

 (obs= 8146)
 Np= 1744 p= 0.21
 Nq= 6402 q= 0.79
 ------------------+------------------+------------------+------------------+
 Coef.= 0.1548 t= 14.1415 P>|t| = 0.0001 df= 8144

 . pbis hispanic bmi if sample1==1 & interview==0

 (obs= 8157)
 Np= 1160 p= 0.14
 Nq= 6997 q= 0.86
 ------------------+------------------+------------------+------------------+
 Coef.= 0.0290 t= 2.6183 P>|t| = 0.0089 df= 8155

 . corr ed bmi if sample1==1 & interview==0
 (obs=8,107)

 | ed bmi
 -------------+------------------
 ed | 1.0000
 bmi | -0.0915 1.0000

S1 Table P displays the correlation between race/ethnicity and our covariates to justify inclusion of race/ethnicity, gender, education, and BMI in the same model. The strongest correlation was between Hispanic race/ethnicity and education (coefficient=-0.384) and does not indicate presence of collinearity between covariates.

NOTE: For ease of interpretation, race/ethnicity was converted to two binary indicator variables (for Non-Hispanic Black and for Hispanic respondents).

**Models: Additional negative binomial models**

Negative binomial models include:

- Model 1: Time (interview) only

- Model 2: Time and race/ethnicity

- Model 3: Time, race/ethnicity, timerace/ethnicity [n=8,331]

- Model 4: Time, race/ethnicity, timerace/ethnicity (IPW) [n=8,267]; Wald test for interaction, p<0.001

- Model 5: Time, race/ethnicity, timerace/ethnicity (IPW trimmed) [n=8,064]; interaction, p<0.001

- Model 6: Time, race/ethnicity, timerace/ethnicity (IPW trimmed X HRS weight) [n=8,064]; interaction, p<0.001

- Model 7: Time, race/ethnicity, timerace/ethnicity, covariates (IPW trimmed) [n=7,811]; interaction, p<0.001

- Model 8: Time, race/ethnicity, timerace/ethnicity, covariates (IPW trimmed X HRS weight) [n=7,811]; interaction, p=0.0014

Covariates include education, gender, and BMI (time-varying). S1 Tables Q and R compare models 3-8, while S1 Table S displays the marginal effect of race/ethnicity on chronic disease at each interview (post-estimation Stata command: *margins*) and S1 Table T displays the effect of time according to race/ethnicity (post-estimation Stata command: *lincom*). Figures G-J display the predicted values from models 5-8. Please refer to the discussion of S1 Table 7 for interpretation of the following model coefficients: intercept, slope, explanatory factors and interactions between explanatory factors and slope.

As stated, S1 Table Q compared models 3-8. These models are presented with and without weights that account for the complex survey design of HRS; probability weights are a respondent’s HRS baseline or entry year survey weight multiplied by our IPW. We were unable to account for the study’s standard error computation unit or stratum as available software will not allow us to create a GEE model while using the survey module.

**GLOBAL PICTURE**: All groups are accumulating disease over time, e.g. the slope is positive. At baseline, Non-Hispanic Black respondents have a higher disease count than Non-Hispanic White and Hispanic respondents. Hispanic respondents are accumulating disease faster than Non-Hispanic White and Non-Hispanic Black respondents. Education and BMI are significant covariates in models that include these factors.

**NOTE ON SIGNIFICANCE OF INTERACTION TERM**: The overall statistical significance of the interaction term (time X race/ethnicity) is list above with model descriptions. For all models, the type III p-value for the interaction term is statistically significant at the 0.01-level.

**NOTE ON SENSITIVITY ANALYSES CONDUCTED:** We explored if the addition of a quadratic or cubic term for time (interview) would improve our model specification. In all models (non-IPW, IPW, covariate adjusted), the interaction between race/ethnicity and quadratic or cubic time was not statistically significant. We also explored the 3-way interactions between time*BMI(continuous and categorical)*race/ethnicity and time*education*race/ethnicity. None of these 3-way interactions were statistically significant at the alpha-level of 0.01. Last, we added net worth into the co-variate adjusted models, which was not statistically significant nor did it change the estimates of other models parameters.

**S1 Table Q.** Comparison of Negative Binominal models 3-8

. esttab model3 model4 model5 model6 model7 model8, se mtitle label constant scalars("chi2")

 --------------------------------------------------------------------------------------------------------------------
 (1) (2) (3) (4) (5) (6)
 model3 model4 model5 model6 model7 model8
 --------------------------------------------------------------------------------------------------------------------
 interview 0.131*** 0.128*** 0.128*** 0.131*** 0.128*** 0.131***
 (0.00208) (0.00191) (0.00189) (0.00214) (0.00194) (0.00220)

 Non-Hispanic White 0 0 0 0 0 0
 (.) (.) (.) (.) (.) (.)

 Non-Hispanic Black 0.329*** 0.336*** 0.335*** 0.333*** 0.261*** 0.246***
 (0.0308) (0.0262) (0.0260) (0.0320) (0.0260) (0.0318)

 Hispanic 0.0216 0.0203 0.0195 0.0115 -0.125** -0.160**
 (0.0423) (0.0357) (0.0344) (0.0434) (0.0384) (0.0486)

 Non-Hispanic White~w 0 0 0 0 0 0
 (.) (.) (.) (.) (.) (.)

 Non-Hispanic Black~w -0.0116** -0.0119** -0.0124*** -0.0133** -0.0109** -0.0109*
 (0.00426) (0.00375) (0.00369) (0.00432) (0.00382) (0.00448)

 Hispanic # interview 0.0147* 0.0242*** 0.0261*** 0.0163** 0.0233*** 0.0147*
 (0.00610) (0.00537) (0.00541) (0.00630) (0.00550) (0.00650)

 Education level -0.0483*** -0.0560***
 (0.00322) (0.00402)

 Gender, female 0.0604*** 0.0456*
 (0.0181) (0.0216)

 BMI 0.0112*** 0.0113***
 (0.000896) (0.00104)

 Constant -0.0191 0.0203 0.00467 -0.0178 0.293*** 0.389***
 (0.0159) (0.0152) (0.0147) (0.0165) (0.0557) (0.0661)
 --------------------------------------------------------------------------------------------------------------------
 Observations 41147 40935 39883 39883 38245 38245
 chi2 5700.1 6757.7 6853.9 5435.2 7079.0 5562.2
 --------------------------------------------------------------------------------------------------------------------
 Standard errors in parentheses
 * p<0.05, ** p<0.01, *** p<0.001

**S1 Table R.** Comparison of Negative Binominal models 3-8, Incident Rate Ratios (IRR; exp coeff)

. esttab model3 model4 model5 model6 model7 model8, se mtitle label constant scalars("chi2") eform

 --------------------------------------------------------------------------------------------------------------------
 (1) (2) (3) (4) (5) (6)
 model3 model4 model5 model6 model7 model8
 --------------------------------------------------------------------------------------------------------------------
 interview 1.140*** 1.137*** 1.136*** 1.140*** 1.137*** 1.140***
 (0.00237) (0.00217) (0.00215) (0.00243) (0.00221) (0.00250)

 Non-Hispanic White 1 1 1 1 1 1
 (.) (.) (.) (.) (.) (.)

 Non-Hispanic Black 1.390*** 1.399*** 1.398*** 1.395*** 1.298*** 1.279***
 (0.0428) (0.0367) (0.0363) (0.0446) (0.0337) (0.0407)

 Hispanic 1.022 1.021 1.020 1.012 0.882** 0.852**
 (0.0432) (0.0364) (0.0350) (0.0439) (0.0339) (0.0414)

 Non-Hispanic White~w 1 1 1 1 1 1
 (.) (.) (.) (.) (.) (.)

 Non-Hispanic Black~w 0.988** 0.988** 0.988*** 0.987** 0.989** 0.989*
 (0.00421) (0.00371) (0.00365) (0.00426) (0.00378) (0.00443)

 Hispanic # interview 1.015* 1.024*** 1.026*** 1.016** 1.024*** 1.015*
 (0.00619) (0.00551) (0.00556) (0.00640) (0.00563) (0.00660)

 Education level 0.953*** 0.946***
 (0.00307) (0.00380)

 Gender, female 1.062*** 1.047*
 (0.0193) (0.0226)

 BMI 1.011*** 1.011***
 (0.000906) (0.00105)

 Constant 0.981 1.021 1.005 0.982 1.340*** 1.476***
 (0.0156) (0.0155) (0.0148) (0.0162) (0.0746) (0.0976)
 --------------------------------------------------------------------------------------------------------------------
 Observations 41147 40935 39883 39883 38245 38245
 chi2 5700.1 6757.7 6853.9 5435.2 7079.0 5562.2
 --------------------------------------------------------------------------------------------------------------------
 Exponentiated coefficients; Standard errors in parentheses
 * p<0.05, ** p<0.01, *** p<0.001

**S1 Table S.** Predicted values: Marginal effects of race/ethnicity on count of diseases, Models 1, 6 & 8^1^

| ***Interview*** | ***Model 1*** | ***Model 6*** | | | ***Model 8*** | | |
| --- | --- | --- | --- | --- | --- | --- | --- |
|  | Overall | NH White | NH Black | Hispanic | NH White | NH Black | Hispanic |
| **1** | 1.026 (0.014) | 0.982 (0.016) | 1.370 (0.038) | 0.994 (0.040) | 0.983 (0.016) | 1.258 (0.034) | 0.838 (0.038) |
| **2** | 1.169 (0.014) | 1.120 (0.017) | 1.541 (0.039) | 1.151 (0.042) | 1.121 (0.017) | 1.418 (0.036) | 0.970 (0.041) |
| **3** | 1.331 (0.015) | 1.276 (0.018) | 1.733 (0.040) | 1.334 (0.044) | 1.279 (0.017) | 1.600 (0.037) | 1.122 (0.043) |
| **4** | 1.516 (0.016) | 1.454 (0.019) | 1.950 (0.042) | 1.545 (0.047) | 1.458 (0.018) | 1.805 (0.040) | 1.299 (0.047) |
| **5** | 1.727 (0.017) | 1.658 (0.020) | 2.193 (0.045) | 1.790 (0.051) | 1.662 (0.020) | 2.036 (0.043) | 1.503 (0.052) |
| **6** | 1.968 (0.019) | 1.889 (0.023) | 2.466 (0.050) | 2.073 (0.059) | 1.896 (0.022) | 2.296 (0.049) | 1.739 (0.059) |
| **7** | 2.241 (0.022) | 2.153 (0.026) | 2.774 (0.057) | 2.402 (0.070) | 2.162 (0.026) | 2.590 (0.057) | 2.013 (0.070) |
| **8** | 2.553 (0.026) | 2.454 (0.031) | 3.120 (0.068) | 2.783 (0.086) | 2.465 (0.031) | 2.921 (0.068) | 2.329 (0.085) |
| **9** | 2.908 (0.032) | 2.797 (0.037) | 3.509 (0.082) | 3.223 (0.109) | 2.811 (0.038) | 3.295 (0.083) | 2.695 (0.105) |

^1^ Values in parentheses are standard errors

**S1 Table T.** Predicted values: Estimated effect of time according to race/ethnicity, Models 1, 6 & 8

|  | ***Model 1*** | ***Model 6*** | ***Model 8*** |
| --- | --- | --- | --- |
| **Overall** | 0.130 (0.002) |  |  |
| **NH White** |  | 0.131 (0.002) | 0.131 (0.002) |
| **NH Black** |  | 0.118 (0.004) | 0.120 (0.004) |
| **Hispanic** |  | 0.147 (0.006) | 0.146 (0.006) |

^1^ Values in parentheses are standard errors


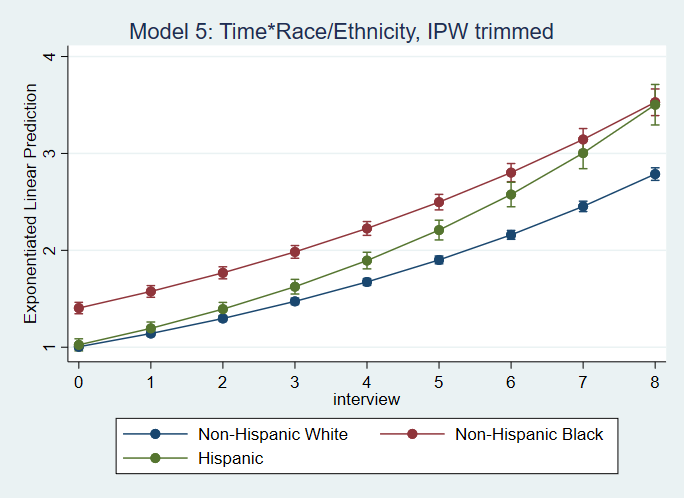


**S1 Figure G**. Model 5: Time by race/ethnicity, IPW trimmed


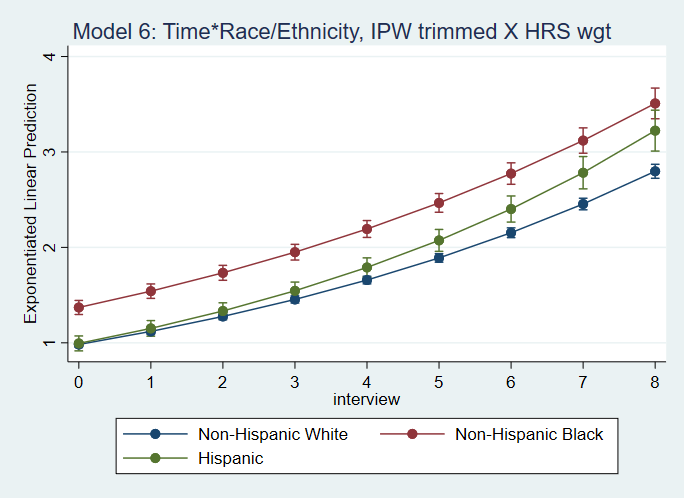


**S1 Figure H**. Model 6: Time by race/ethnicity, IPW trimmed, HRS weights


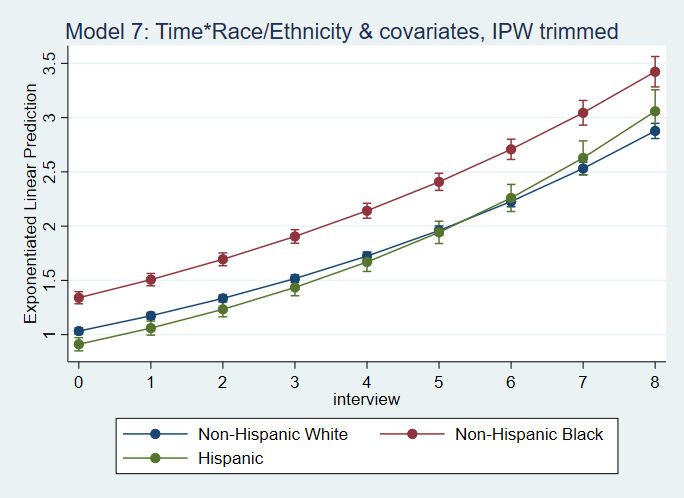


**S1 Figure I**. Model 7: Time by race/ethnicity and covariates, IPW trimmed


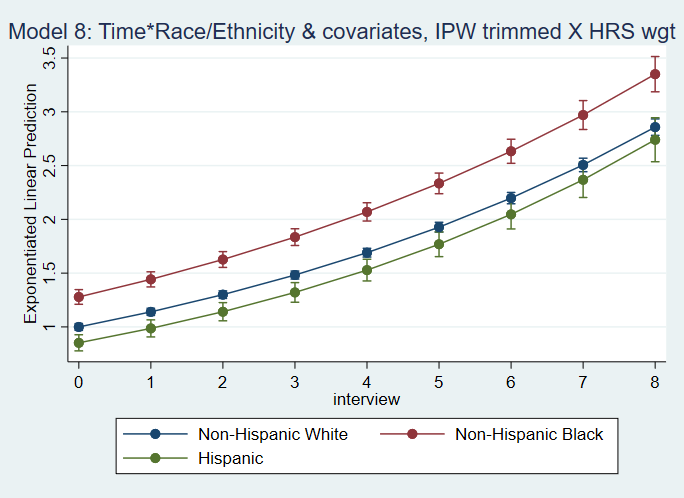


**S1 Figure J**. Model 8: Time by race/ethnicity and covariates, IPW trimmed, HRS weights
